# Supplementary material for: Momentary assessment of parent and child emotion regulation to inform the design of a new emotion-focused parenting app
Source: PLoS One. 2025 Jul 3;20(7):e0327179. doi: 10.1371/journal.pone.0327179 (PMC12225822; doi:10.1371/journal.pone.0327179)
Supplement: S1 Table — (DOCX) [file pone.0327179.s001.docx]

**S1 Table. Short survey prompts schedules.**

| Schedule 1 (*n* = 48) | Schedule 2 (*n* = 41) |
| --- | --- |
| 6:30am | 7:30am |
| 9:00am | 11:00am |
| 1:00pm | 2:30pm |
| 4:30pm | 6:00pm |
| 7:30pm | 9:00pm |
