# Supplementary material for: Momentary assessment of parent and child emotion regulation to inform the design of a new emotion-focused parenting app
Source: PLoS One. 2025 Jul 3;20(7):e0327179. doi: 10.1371/journal.pone.0327179 (PMC12225822; doi:10.1371/journal.pone.0327179)
Supplement: S2 Table — (DOCX) [file pone.0327179.s002.docx]

**S2 Table. Short survey response options for difficult parenting situations arranged by theme.**

| Parenting Situation |
| --- |
| Food/Mealtimes |
| Struggling to get the child to sit down for mealtime |
| Child whining for a treat |
| Child being fussy about food |
| Bedtime routine |
| Child resisting brushing their teeth |
| Child resisting a bath/shower |
| Child resisting going to bed/sleep |
| Child complaining that they’re hungry/thirsty |
| Child not staying in bed |
| Siblings |
| Child fighting with their sibling |
| Child refusing to play/interact with their sibling |
| Screen time |
| Child having a meltdown when the screen was turned off |
| Child was whining for (more) screen time |
| Getting dressed |
| Child was resisting getting dressed |
| Child insisting on inappropriate outfit |
| Child not accepting help to dress |
| Transitions/Leaving the house |
| Child refusing to stop activity |
| Child not putting shoes on |
| Child not wanting to leave my side/refusing to play |
| Child struggling to leave other parent |
| Safety and danger |
| Child refusing hat/sunscreen/sunsafe clothing |
| Child not holding hands whilst crossing road |
| Child walking/scooting/riding out of sight |
| Child resisting car seatbelt/harness |
| Car travel |
| Child resisting getting in/out of the car |
| Child whining for food |
| Child insisting on opening doors/windows |
| Child and sibling(s) fighting in the car |
