# Supplementary material for: Momentary assessment of parent and child emotion regulation to inform the design of a new emotion-focused parenting app
Source: PLoS One. 2025 Jul 3;20(7):e0327179. doi: 10.1371/journal.pone.0327179 (PMC12225822; doi:10.1371/journal.pone.0327179)
Supplement: S3 Table — (DOCX) [file pone.0327179.s003.docx]

**S3 Table. Individual adult PANAS item regression results with unstandardised coefficients and 95% confidence intervals.**

| Item | *B* | 95% CI | | *p* |
| --- | --- | --- | --- | --- |
|  |  | *LL* | *UL* |  |
| Upset | 2.13 | 2.07 | 2.20 | <0.001 |
| Hostile | 2.50 | 2.39 | 2.62 | <0.001 |
| Ashamed | 2.81 | 2.69 | 2.94 | <0.001 |
| Nervous | 1.92 | 1.83 | 2.00 | <0.001 |
| Afraid | 2.90 | 2.74 | 3.05 | <0.001 |

CI = confidence interval; LL = lower limit; UL = upper limit.
