# Supplementary material for: Momentary assessment of parent and child emotion regulation to inform the design of a new emotion-focused parenting app
Source: PLoS One. 2025 Jul 3;20(7):e0327179. doi: 10.1371/journal.pone.0327179 (PMC12225822; doi:10.1371/journal.pone.0327179)
Supplement: S4 Table — (DOCX) [file pone.0327179.s004.docx]

**S4 Table. Individual child PANAS item regression results with unstandardised coefficients and 95% confidence intervals.**

| Item | *B* | 95% CI | | *p* |
| --- | --- | --- | --- | --- |
|  |  | *LL* | *UL* |  |
| Depressed | 3.80 | 3.47 | 4.14 | <0.001 |
| Angry | 1.88 | 1.78 | 1.98 | <0.001 |
| Scared | 2.96 | 2.80 | 3.12 | <0.001 |
| Afraid | 3.11 | 2.92 | 3.30 | <0.001 |
| Sad | 1.82 | 1.75 | 1.89 | <0.001 |

CI = confidence interval; LL = lower limit; UL = upper limit.
