# Supplementary material for: Momentary assessment of parent and child emotion regulation to inform the design of a new emotion-focused parenting app
Source: PLoS One. 2025 Jul 3;20(7):e0327179. doi: 10.1371/journal.pone.0327179 (PMC12225822; doi:10.1371/journal.pone.0327179)
Supplement: S5 Table — (DOCX) [file pone.0327179.s005.docx]

**S5 Table. Individual adult S-DERS item regression results with unstandardised coefficients and 95% confidence intervals.**

| Item | *B* | 95% CI | | *p* |
| --- | --- | --- | --- | --- |
|  |  | *LL* | *UL* |  |
| My emotions feel overwhelming | 2.11 | 2.01 | 2.20 | <0.001 |
| I am having difficulty controlling my behaviours | 2.66 | 2.53 | 2.80 | <0.001 |
| I am having difficulty doing the things I need to do right now | 1.72 | 1.64 | 1.79 | <0.001 |
| I am paying attention to how I feel | 0.85 | 0.77 | 0.93 | <0.001 |
| I have no idea how I am feeling | 1.63 | 1.52 | 1.74 | <0.001 |

CI = confidence interval; LL = lower limit; UL = upper limit.
