# Supplementary material for: Momentary assessment of parent and child emotion regulation to inform the design of a new emotion-focused parenting app
Source: PLoS One. 2025 Jul 3;20(7):e0327179. doi: 10.1371/journal.pone.0327179 (PMC12225822; doi:10.1371/journal.pone.0327179)
Supplement: S6 Table — (DOCX) [file pone.0327179.s006.docx]

**S6 Table. Individual child S-DERS item regression results with unstandardised coefficients and 95% confidence intervals.**

| Item | *B* | 95% CI | | *p* |
| --- | --- | --- | --- | --- |
|  |  | *LL* | *UL* |  |
| My child seems to be overwhelmed by their emotions | 3.13 | 3.04 | 3.22 | <0.001 |
| My child seems to be feeling out of control | 3.68 | 3.58 | 3.79 | <0.001 |
| My child is having difficulty controlling their behaviours | 3.19 | 3.11 | 3.26 | <0.001 |
| My child is having difficulty doing the things they need to do right now | 2.58 | 2.49 | 2.66 | <0.001 |

CI = confidence interval; LL = lower limit; UL = upper limit.
