# Supplementary material for: Momentary assessment of parent and child emotion regulation to inform the design of a new emotion-focused parenting app
Source: PLoS One. 2025 Jul 3;20(7):e0327179. doi: 10.1371/journal.pone.0327179 (PMC12225822; doi:10.1371/journal.pone.0327179)
Supplement: S7 Table — (DOCX) [file pone.0327179.s007.docx]

**S7 Table. Association of individual parent PANAS short survey items with baseline measures and subscales.**

| Baseline measure | Parent PANAS items, *B* (95% CI [*LL, UL*]) | | | | |
| --- | --- | --- | --- | --- | --- |
|  | Item 1 (Upset) | Item 2 (Hostile) | Item 3 (Ashamed) | Item 4 (Nervous) | Item 5 (Afraid) |
| Negative affect | 0.17 (0.01, 0.33)* | 0.16 (0.02, 0.31)* | 0.08 (-0.04, 0.20) | 0.35 (0.06, 0.64)* | 0.13 (-0.02, 0.28) |
| SMFQ | -0.01 (-0.07, 0.05) | -0.01 (-0.06, 0.05) | 0.01 (-0.04, 0.05) | 0.04 (-0.07, 0.15) | 0.00 (-0.05, 0.06) |
| SCAS | 0.00 (-0.08, 0.08) | 0.00 (-0.07, 0.08) | 0.02 (-0.04, 0.08) | 0.18 (0.03, 0.32)* | 0.05 (-0.03, 0.12) |
| SNAP | 0.02 (-0.03, 0.06) | 0.02 (-0.02, 0.06) | 0.01 (-0.02, 0.04) | 0.13 (0.06, 0.20)*** | 0.01 (-0.02, 0.05) |
| STSC (Sociability) | -0.09 (-0.25, 0.08) | -0.05 (-0.21, 0.10) | -0.04 (-0.17, 0.08) | -0.13 (-0.44, 0.17) | -0.02 (-0.18, 0.14) |
| STSC (Persistence) | -0.01 (-0.19, 0.16) | 0.05 (-0.12, 0.21) | 0.04 (-0.09, 0.18) | -0.16 (-0.48, 0.16) | 0.01 (-0.15, 0.18) |
| PRFQ (Pre-mentalising) | 0.07 (-0.12, 0.25) | -0.06 (-0.23, 0.11) | -0.01 (-0.15, 0.13) | -0.09 (-0.43, 0.25) | -0.02 (-0.20, 0.15) |
| PRFQ (Certainty) | -0.01 (-0.15, 0.13) | 0.02 (-0.10, 0.15) | -0.01 (-0.11, 0.09) | -0.06 (-0.30, 0.19) | 0.00 (-0.12, 0.13) |
| PRFQ (Interest) | -0.13 (-0.38, 0.12) | -0.11 (-0.34, 0.12) | 0.04 (-0.14, 0.22) | 0.12 (-0.33, 0.57) | 0.15 (-0.09, 0.38) |
| PBACE (Manipulation) | -0.02 (-0.05, 0.01) | 0.00 (-0.03, 0.03) | -0.02 (-0.04, 0.00) | -0.06 (-0.12, -0.01)* | -0.03 (-0.06, 0.00) |
| PBACE (Autonomy) | -0.02 (-0.05, 0.00) | -0.02 (-0.04, 0.01) | -0.02 (-0.04, 0.00) | -0.07 (-0.11, -0.02)** | -0.03 (-0.06, -0.01)* |
| PBACE (Stability) | -0.01 (-0.06, 0.04) | 0.00 (-0.05, 0.04) | 0.01 (-0.03, 0.05) | 0.07 (-0.02, 0.16) | 0.04 (-0.01, 0.09) |
| PBACE (Anger) | 0.00 (-0.04, 0.04) | -0.01 (-0.04, 0.03) | 0.00 (-0.03, 0.02) | 0.01 (-0.06, 0.08) | 0.02 (-0.02, 0.05) |
| PBACE (Control) | -0.02 (-0.06, 0.01) | 0.00 (-0.03, 0.04) | -0.02 (-0.05, 0.00) | -0.12 (-0.18, -0.05)*** | -0.04 (-0.08, -0.01)* |
| SEFQ (Negative) | 0.14 (0.03, 0.25)* | 0.05 (-0.06, 0.15) | 0.07 (-0.01, 0.16) | 0.22 (0.02, 0.43)* | 0.13 (0.03, 0.24)* |
| SEFQ (Positive) | -0.03 (-0.15, 0.08) | 0.00 (-0.10, 0.11) | -0.02 (-0.11, 0.06) | 0.01 (-0.20, 0.21) | -0.03 (-0.14, 0.08) |
| DERS (Total) | 0.01 (0.00, 0.02)* | 0.01 (0.00, 0.02)* | 0.02 (0.01, 0.02)*** | 0.02 (0.01, 0.04)** | 0.01 (0.00, 0.02)** |
| DERS (Non-acceptance) | 0.04 (-0.01, 0.09) | 0.02 (-0.02, 0.06) | 0.08 (0.05, 0.11)*** | 0.11 (0.03, 0.20)** | 0.07 (0.02, 0.11)** |
| DERS (Goal-directed) | 0.05 (0.00, 0.10)* | 0.05 (0.01, 0.10)* | 0.04 (0.01, 0.08)* | 0.13 (0.04, 0.21)** | 0.06 (0.02, 0.11)** |
| DERS (Impulsivity) | 0.04 (0.01, 0.07)** | 0.03 (0.01, 0.06)* | 0.04 (0.02, 0.06)*** | 0.06 (0.01, 0.12)* | 0.04 (0.01, 0.06)** |
| DERS (Strategies) | 0.03 (0.00, 0.06)* | 0.03 (0.01, 0.06)* | 0.04 (0.02, 0.06)*** | 0.06 (0.00, 0.11)* | 0.03 (0.00, 0.06)* |
| DERS (Clarity) | 0.06 (-0.04, 0.16) | 0.04 (-0.05, 0.13) | 0.11 (0.04, 0.18)** | 0.17 (-0.01, 0.35) | 0.07 (-0.02, 0.17) |
| Kessler-6 | 0.05 (0.01, 0.08)** | 0.03 (0.00, 0.06) | 0.04 (0.01, 0.06)** | 0.12 (0.06, 0.17)*** | 0.06 (0.03, 0.09)*** |
| PANAS | 0.01 (-0.04, 0.05) | 0.00 (-0.04, 0.04) | 0.00 (-0.03, 0.03) | 0.00 (-0.08, 0.08) | 0.02 (-0.02, 0.06) |
| DASS (Stress) | 0.02 (0.00, 0.03) | 0.01 (-0.01, 0.03) | 0.02 (0.01, 0.03)** | 0.06 (0.03, 0.09)*** | 0.03 (0.01, 0.04)** |
| Verbal partner conflict | 0.36 (0.00, 0.72) | 0.24 (-0.08, 0.56) | 0.28 (-0.01, 0.56) | 1.04 (0.32, 1.75)** | 0.43 (0.05, 0.82)* |
| Physical partner conflict | 1.06 (0.10, 2.03)* | 0.23 (-0.64, 1.1) | 0.12 (-0.66, 0.91) | -0.25 (-2.28, 1.79) | 0.28 (-0.79, 1.35) |

* = *p*<0.05; ** = *p*<0.01; *** = *p*<0.001
