# Supplementary material for: Momentary assessment of parent and child emotion regulation to inform the design of a new emotion-focused parenting app
Source: PLoS One. 2025 Jul 3;20(7):e0327179. doi: 10.1371/journal.pone.0327179 (PMC12225822; doi:10.1371/journal.pone.0327179)
Supplement: S8 Table — (DOCX) [file pone.0327179.s008.docx]

**S8 Table. Association of individual child PANAS short survey items with baseline measures and subscales.**

| Baseline measure | Child PANAS items, *B* (95% CI [*LL, UL*]) | | | | |
| --- | --- | --- | --- | --- | --- |
|  | Item 1 (Depressed) | Item 2 (Angry) | Item 3 (Scared) | Item 4 (Afraid) | Item 5 (Sad) |
| Negative affect | 0.08 (0.02, 0.13)** | 0.38 (0.22, 0.54)*** | 0.14 (0.06, 0.22)*** | 0.11 (0.04, 0.18)** | 0.19 (0.03, 0.35)* |
| SMFQ | 0.01 (-0.01, 0.03) | 0.05 (-0.01, 0.11) | 0.02 (-0.01, 0.05) | 0.02 (-0.01, 0.04) | 0.02 (-0.04, 0.08) |
| SCAS | 0.03 (0.00, 0.06) | 0.07 (-0.01, 0.16) | 0.04 (0.00, 0.09)* | 0.04 (0.01, 0.08)* | 0.03 (-0.05, 0.11) |
| SNAP | 0.01 (-0.01, 0.02) | 0.05 (0.01, 0.09)* | 0.02 (0.00, 0.04) | 0.01 (0.00, 0.03) | 0.04 (0.00, 0.08) |
| STSC (Sociability) | -0.08 (-0.13, -0.02)** | -0.17 (-0.35, 0.01) | -0.08 (-0.17, 0.00) | -0.05 (-0.13, 0.02) | -0.09 (-0.26, 0.08) |
| STSC (Persistence) | 0.00 (-0.07, 0.06) | -0.09 (-0.28, 0.11) | -0.06 (-0.16, 0.03) | -0.03 (-0.11, 0.05) | -0.25 (-0.42, -0.08)** |
| PRFQ (Pre-mentalising) | 0.07 (0.00, 0.13)* | 0.15 (-0.05, 0.35) | 0.10 (0.00, 0.19)* | 0.08 (0.00, 0.16)* | -0.03 (-0.22, 0.16) |
| PRFQ (Certainty) | -0.01 (-0.06, 0.04) | 0.00 (-0.15, 0.15) | 0.01 (-0.07, 0.08) | 0.01 (-0.05, 0.07) | 0.02 (-0.12, 0.16) |
| PRFQ (Interest) | 0.06 (-0.02, 0.15) | 0.03 (-0.24, 0.30) | 0.04 (-0.09, 0.17) | 0.02 (-0.09, 0.13) | 0.06 (-0.19, 0.31) |
| PBACE (Manipulation) | 0.01 (-0.01, 0.02) | 0.03 (-0.01, 0.06) | 0.00 (-0.01, 0.02) | 0.00 (-0.01, 0.02) | -0.02 (-0.05, 0.01) |
| PBACE (Autonomy) | 0.00 (-0.01, 0.01) | -0.02 (-0.05, 0.01) | -0.01 (-0.03, 0.00) | -0.01 (-0.02, 0.00) | -0.04 (-0.06, -0.01)** |
| PBACE (Stability) | 0.01 (-0.01, 0.03) | 0.02 (-0.04, 0.07) | 0.02 (-0.01, 0.05) | 0.02 (-0.01, 0.04) | 0.03 (-0.03, 0.08) |
| PBACE (Anger) | 0.00 (-0.01, 0.01) | -0.01 (-0.04, 0.03) | 0.00 (-0.02, 0.02) | 0.00 (-0.01, 0.02) | 0.01 (-0.02, 0.05) |
| PBACE (Control) | 0.01 (-0.01, 0.02) | 0.00 (-0.04, 0.05) | -0.01 (-0.03, 0.01) | -0.01 (-0.03, 0.01) | -0.03 (-0.07, 0.01) |
| SEFQ (Negative) | 0.03 (-0.01, 0.07) | 0.22 (0.10, 0.33)*** | 0.08 (0.03, 0.14)** | 0.10 (0.05, 0.15)*** | 0.09 (-0.02, 0.20) |
| SEFQ (Positive) | -0.02 (-0.06, 0.02) | -0.01 (-0.13, 0.12) | -0.03 (-0.09, 0.03) | -0.03 (-0.09, 0.02) | -0.05 (-0.17, 0.06) |
| DERS (Total) | 0.00 (0.00, 0.01)* | 0.01 (0.00, 0.02) | 0.00 (0.00, 0.01) | 0.01 (0.00, 0.01)** | 0.00 (-0.01, 0.01) |
| DERS (Non-acceptance) | 0.02 (0.00, 0.03) | -0.01 (-0.06, 0.05) | 0.02 (-0.01, 0.04) | 0.03 (0.01, 0.05)* | -0.02 (-0.06, 0.03) |
| DERS (Goal-directed) | 0.02 (0.00, 0.03)* | 0.04 (-0.01, 0.09) | 0.03 (0.00, 0.05)* | 0.02 (0.00, 0.05)* | 0.03 (-0.02, 0.08) |
| DERS (Impulsivity) | 0.01 (0.00, 0.02)* | 0.03 (0.00, 0.07)* | 0.01 (0.00, 0.03) | 0.02 (0.00, 0.03)** | 0.00 (-0.03, 0.03) |
| DERS (Strategies) | 0.01 (0.00, 0.02)* | 0.02 (-0.02, 0.05) | 0.01 (-0.01, 0.03) | 0.01 (0.00, 0.03)* | 0.00 (-0.04, 0.03) |
| DERS (Clarity) | 0.05 (0.01, 0.08)** | 0.06 (-0.05, 0.17) | 0.06 (0.01, 0.11)* | 0.06 (0.01, 0.10)* | 0.03 (-0.07, 0.13) |
| Kessler-6 | 0.01 (0.00, 0.02) | 0.05 (0.01, 0.08)** | 0.02 (0.00, 0.04)* | 0.02 (0.00, 0.03)** | 0.02 (-0.02, 0.05) |
| PANAS | 0.00 (-0.01, 0.02) | 0.01 (-0.04, 0.06) | 0.00 (-0.02, 0.02) | 0.00 (-0.02, 0.02) | 0.00 (-0.05, 0.04) |
| DASS (Stress) | 0.00 (0.00, 0.01) | 0.02 (0.00, 0.04)* | 0.01 (0.00, 0.02)* | 0.01 (0.01, 0.02)*** | 0.02 (0.00, 0.04) |
| Verbal partner conflict | 0.08 (-0.06, 0.22) | 0.59 (0.18, 1.00)** | 0.37 (0.20, 0.54)*** | 0.38 (0.22, 0.54)*** | 0.26 (-0.15, 0.67) |
| Physical partner conflict | 0.32 (-0.05, 0.70) | 1.52 (0.41, 2.62)** | 0.73 (0.24, 1.23)** | 0.46 (-0.02, 0.93) | -0.15 (-1.26, 0.96) |

* = *p*<0.05; ** = *p*<0.01; *** = *p*<0.001
