# Supplementary material for: Momentary assessment of parent and child emotion regulation to inform the design of a new emotion-focused parenting app
Source: PLoS One. 2025 Jul 3;20(7):e0327179. doi: 10.1371/journal.pone.0327179 (PMC12225822; doi:10.1371/journal.pone.0327179)
Supplement: S9 Table — (DOCX) [file pone.0327179.s009.docx]

**S9 Table. Association of individual parent S-DERS short survey items with baseline measures and subscales.**

| Baseline measure | Parent S-DERS items, *B* (95% CI [*LL, UL*]) | | | | |
| --- | --- | --- | --- | --- | --- |
|  | Item 1^a^ | Item 2^b^ | Item 3^c^ | Item 4^d^ | Item 5^e^ |
| Negative affect | 0.40 (0.16, 0.64)** | 0.21 (0.05, 0.37)* | 0.54 (0.27, 0.81)*** | 0.20 (-0.30, 0.70) | -0.08 (-0.40, 0.23) |
| SMFQ | 0.03 (-0.07, 0.12) | 0.00 (-0.07, 0.06) | 0.05 (-0.06, 0.16) | 0.08 (-0.10, 0.26) | -0.06 (-0.18, 0.06) |
| SCAS | 0.10 (-0.03, 0.22) | 0.03 (-0.05, 0.12) | 0.18 (0.04, 0.32)* | 0.05 (-0.20, 0.30) | -0.03 (-0.19, 0.13) |
| SNAP | 0.08 (0.02, 0.14)* | 0.02 (-0.02, 0.06) | 0.13 (0.07, 0.20)*** | 0.11 (-0.01, 0.23) | -0.02 (-0.10, 0.06) |
| STSC (Sociability) | -0.18 (-0.44, 0.07) | -0.07 (-0.24, 0.10) | -0.20 (-0.49, 0.10) | -0.08 (-0.59, 0.42) | 0.22 (-0.10, 0.55) |
| STSC (Persistence) | -0.04 (-0.31, 0.24) | 0.07 (-0.12, 0.25) | -0.03 (-0.34, 0.29) | -0.30 (-0.84, 0.23) | 0.13 (-0.22, 0.47) |
| PRFQ (Pre-mentalising) | 0.30 (0.02, 0.58)* | 0.21 (0.03, 0.40)* | 0.05 (-0.28, 0.39) | 0.41 (-0.15, 0.97) | 0.05 (-0.31, 0.42) |
| PRFQ (Certainty) | 0.02 (-0.19, 0.23) | 0.05 (-0.09, 0.19) | 0.08 (-0.16, 0.32) | 0.14 (-0.27, 0.55) | -0.01 (-0.28, 0.25) |
| PRFQ (Interest) | -0.05 (-0.43, 0.33) | -0.03 (-0.29, 0.23) | -0.07 (-0.52, 0.37) | -0.51 (-1.26, 0.23) | 0.09 (-0.39, 0.57) |
| PBACE (Manipulation) | -0.02 (-0.07, 0.02) | -0.01 (-0.04, 0.02) | -0.03 (-0.09, 0.02) | 0.03 (-0.06, 0.13) | -0.02 (-0.09, 0.04) |
| PBACE (Autonomy) | -0.04 (-0.08, 0.00) | -0.02 (-0.05, 0.01) | -0.04 (-0.09, 0.00) | 0.05 (-0.03, 0.13) | 0.00 (-0.05, 0.05) |
| PBACE (Stability) | 0.04 (-0.04, 0.12) | -0.01 (-0.06, 0.05) | 0.07 (-0.03, 0.16) | 0.01 (-0.15, 0.16) | -0.01 (-0.11, 0.10) |
| PBACE (Anger) | -0.01 (-0.07, 0.04) | -0.03 (-0.06, 0.01) | -0.02 (-0.09, 0.04) | -0.06 (-0.17, 0.05) | 0.01 (-0.06, 0.08) |
| PBACE (Control) | -0.04 (-0.10, 0.02) | -0.02 (-0.06, 0.02) | -0.05 (-0.12, 0.02) | 0.12 (0.01, 0.24)* | 0.01 (-0.06, 0.09) |
| SEFQ (Negative) | 0.27 (0.11, 0.44)** | 0.17 (0.06, 0.28)** | 0.29 (0.09, 0.48)** | 0.04 (-0.31, 0.38) | 0.11 (-0.12, 0.33) |
| SEFQ (Positive) | -0.03 (-0.21, 0.14) | -0.01 (-0.13, 0.11) | -0.03 (-0.23, 0.17) | -0.43 (-0.76, -0.10)* | -0.03 (-0.25, 0.19) |
| DERS (Total) | 0.03 (0.01, 0.04)*** | 0.02 (0.01, 0.03)*** | 0.03 (0.02, 0.05)*** | 0.02 (-0.01, 0.05) | 0.02 (0.00, 0.04)* |
| DERS (Non-acceptance) | 0.10 (0.03, 0.17)** | 0.08 (0.03, 0.12)** | 0.13 (0.05, 0.21)** | 0.11 (-0.03, 0.26) | 0.10 (0.01, 0.19)* |
| DERS (Goal-directed) | 0.12 (0.05, 0.19)*** | 0.08 (0.03, 0.12)** | 0.16 (0.08, 0.24)*** | 0.09 (-0.05, 0.24) | 0.04 (-0.06, 0.13) |
| DERS (Impulsivity) | 0.09 (0.04, 0.13)*** | 0.06 (0.03, 0.09)*** | 0.10 (0.04, 0.15)*** | 0.01 (-0.08, 0.11) | 0.08 (0.02, 0.14)** |
| DERS (Strategies) | 0.07 (0.02, 0.11)** | 0.05 (0.03, 0.08)*** | 0.09 (0.04, 0.14)*** | 0.07 (-0.02, 0.16) | 0.04 (-0.02, 0.10) |
| DERS (Clarity) | 0.13 (-0.02, 0.28) | 0.14 (0.04, 0.24)** | 0.19 (0.01, 0.36)* | 0.10 (-0.21, 0.41) | 0.22 (0.03, 0.41)* |
| Kessler-6 | 0.11 (0.06, 0.15)*** | 0.06 (0.03, 0.09)*** | 0.11 (0.06, 0.17)*** | 0.01 (-0.09, 0.11) | 0.04 (-0.02, 0.11) |
| PANAS | 0.03 (-0.04, 0.09) | 0.02 (-0.02, 0.07) | -0.02 (-0.10, 0.05) | -0.11 (-0.23, 0.02) | 0.03 (-0.05, 0.11) |
| DASS (Stress) | 0.05 (0.02, 0.07)*** | 0.02 (0.00, 0.04)* | 0.07 (0.03, 0.10)*** | 0.05 (0.00, 0.11) | 0.03 (0.00, 0.07) |
| Verbal partner conflict | 0.99 (0.45, 1.53)*** | 0.70 (0.37, 1.04)*** | 1.26 (0.60, 1.92)*** | 0.44 (-0.71, 1.59) | 0.41 (-0.38, 1.20) |
| Physical partner conflict | 2.08 (0.57, 3.58)** | 2.01 (1.11, 2.91)*** | 0.91 (-1.03, 2.85) | -0.12 (-3.24, 2.99) | 0.90 (-1.24, 3.03) |

* = *p*<0.05; ** = *p*<0.01; *** = *p*<0.001

^a^ S-DERS Item 1 – My emotions feel overwhelming

^b^ S-DERS Item 2 – I am having difficulty controlling my behaviours

^c^ S-DERS Item 3 – I am having difficulty doing the things I need to do right now

^d^ S-DERS Item 4 – I am paying attention to how I feel

^e^ S-DERS Item 5 – I have no idea how I am feeling
