# Supplementary material for: Momentary assessment of parent and child emotion regulation to inform the design of a new emotion-focused parenting app
Source: PLoS One. 2025 Jul 3;20(7):e0327179. doi: 10.1371/journal.pone.0327179 (PMC12225822; doi:10.1371/journal.pone.0327179)
Supplement: S10 Table — (DOCX) [file pone.0327179.s010.docx]

**S10 Table. Association of individual child S-DERS short survey items with baseline measures and subscales.**

| Baseline measure | Child S-DERS items, *B* (95% CI [*LL, UL*]) | | | |
| --- | --- | --- | --- | --- |
|  | Item 1^a^ | Item 2^b^ | Item 3^c^ | Item 4^d^ |
| Negative affect | 0.37 (0.18, 0.55)*** | 0.24 (0.08, 0.40)** | 0.32 (0.11, 0.53)** | 0.35 (0.09, 0.61)** |
| SMFQ | 0.04 (-0.04, 0.11) | 0.02 (-0.04, 0.08) | 0.02 (-0.06, 0.10) | 0.03 (-0.07, 0.13) |
| SCAS | 0.10 (0.00, 0.20)* | 0.07 (-0.01, 0.15) | 0.06 (-0.05, 0.17) | 0.01 (-0.13, 0.15) |
| SNAP | 0.05 (0.00, 0.10)* | 0.05 (0.01, 0.09)* | 0.06 (0.00, 0.11)* | 0.07 (0.00, 0.14)* |
| STSC (Sociability) | -0.13 (-0.33, 0.08) | -0.09 (-0.26, 0.09) | -0.07 (-0.29, 0.16) | -0.03 (-0.31, 0.25) |
| STSC (Persistence) | -0.13 (-0.34, 0.09) | -0.06 (-0.24, 0.12) | -0.03 (-0.27, 0.20) | 0.04 (-0.26, 0.33) |
| PRFQ (Pre-mentalising) | 0.31 (0.10, 0.53)** | 0.22 (0.04, 0.41)* | 0.19 (-0.06, 0.44) | 0.17 (-0.14, 0.48) |
| PRFQ (Certainty) | 0.04 (-0.13, 0.20) | 0.02 (-0.12, 0.16) | -0.08 (-0.26, 0.11) | -0.12 (-0.34, 0.11) |
| PRFQ (Interest) | 0.14 (-0.16, 0.44) | 0.09 (-0.16, 0.35) | 0.00 (-0.33, 0.34) | -0.11 (-0.52, 0.30) |
| PBACE (Manipulation) | 0.02 (-0.02, 0.06) | 0.01 (-0.02, 0.04) | 0.02 (-0.02, 0.06) | 0.02 (-0.03, 0.08) |
| PBACE (Autonomy) | -0.03 (-0.06, 0.00) | -0.02 (-0.05, 0.00) | -0.03 (-0.06, 0.01) | -0.03 (-0.08, 0.01) |
| PBACE (Stability) | 0.02 (-0.04, 0.09) | 0.03 (-0.03, 0.08) | 0.03 (-0.04, 0.10) | 0.04 (-0.04, 0.13) |
| PBACE (Anger) | 0.00 (-0.04, 0.05) | -0.02 (-0.06, 0.01) | -0.02 (-0.07, 0.03) | -0.03 (-0.09, 0.03) |
| PBACE (Control) | -0.01 (-0.06, 0.04) | -0.02 (-0.06, 0.02) | -0.02 (-0.07, 0.04) | -0.02 (-0.09, 0.04) |
| SEFQ (Negative) | 0.26 (0.13, 0.39)*** | 0.20 (0.10, 0.31)*** | 0.26 (0.12, 0.41)*** | 0.27 (0.09, 0.45)** |
| SEFQ (Positive) | 0.02 (-0.12, 0.16) | -0.04 (-0.16, 0.08) | -0.03 (-0.18, 0.12) | -0.12 (-0.30, 0.07) |
| DERS (Total) | 0.01 (-0.01, 0.02) | 0.01 (0.00, 0.02) | 0.01 (0.00, 0.02) | 0.02 (0.01, 0.04)** |
| DERS (Non-acceptance) | 0.01 (-0.05, 0.07) | 0.03 (-0.02, 0.08) | 0.01 (-0.05, 0.07) | 0.05 (-0.03, 0.13) |
| DERS (Goal-directed) | 0.03 (-0.03, 0.09) | 0.04 (-0.01, 0.09) | 0.04 (-0.03, 0.10) | 0.09 (0.02, 0.17)* |
| DERS (Impulsivity) | 0.03 (-0.01, 0.06) | 0.02 (-0.01, 0.05) | 0.04 (0.00, 0.08) | 0.08 (0.03, 0.12)** |
| DERS (Strategies) | 0.01 (-0.03, 0.05) | 0.02 (-0.02, 0.05) | 0.03 (-0.01, 0.07) | 0.07 (0.02, 0.11)** |
| DERS (Clarity) | 0.06 (-0.06, 0.18) | 0.07 (-0.03, 0.17) | 0.07 (-0.07, 0.20) | 0.10 (-0.06, 0.27) |
| Kessler-6 | 0.04 (0.00, 0.08)* | 0.04 (0.00, 0.07)* | 0.05 (0.01, 0.10)* | 0.07 (0.02, 0.13)** |
| PANAS | 0.04 (-0.01, 0.09) | 0.02 (-0.02, 0.06) | 0.04 (-0.01, 0.10) | 0.02 (-0.05, 0.09) |
| DASS (Stress) | 0.03 (0.01, 0.05)* | 0.03 (0.01, 0.05)** | 0.03 (0.01, 0.05)* | 0.04 (0.01, 0.07)** |
| Verbal partner conflict | 0.93 (0.50, 1.37)*** | 0.79 (0.43, 1.15)*** | 1.04 (0.57, 1.50)*** | 1.11 (0.59, 1.64)*** |
| Physical partner conflict | 1.99 (0.73, 3.25)** | 1.11 (0.01, 2.22)* | 0.94 (-0.50, 2.39) | 0.54 (-1.10, 2.18) |

* = *p*<0.05; ** = *p*<0.01; *** = *p*<0.001

^a^ S-DERS Item 1 – My child seems to be overwhelmed by their emotions

^b^ S-DERS Item 2 – My child seems to be feeling out of control

^c^ S-DERS Item 3 – My child is having difficulty controlling their behaviours

^d^ S-DERS Item 4 – My child is having difficulty doing the things they need to do right now
