# Supplementary material for: Momentary assessment of parent and child emotion regulation to inform the design of a new emotion-focused parenting app
Source: PLoS One. 2025 Jul 3;20(7):e0327179. doi: 10.1371/journal.pone.0327179 (PMC12225822; doi:10.1371/journal.pone.0327179)
Supplement: S11 Table — (DOCX) [file pone.0327179.s011.docx]

**S11 Table. Association of individual parent PANAS short survey items with other short survey items.**

| Short survey item | Parent PANAS items, *B* (95% CI [*LL, UL*]) | | | | |
| --- | --- | --- | --- | --- | --- |
|  | Item 1 (Upset) | Item 2 (Hostile) | Item 3 (Ashamed) | Item 4 (Nervous) | Item 5 (Afraid) |
| Parent PANAS 1 |  | 0.40 (0.38, 0.43)*** | 0.29 (0.27, 0.32)*** | 0.28 (0.24, 0.31)*** | 0.17 (0.15, 0.20)*** |
| Parent PANAS 2 | 0.76 (0.71, 0.80)*** |  | 0.37 (0.34, 0.41)*** | 0.24 (0.18, 0.29)*** | 0.14 (0.11, 0.17)*** |
| Parent PANAS 3 | 0.69 (0.63, 0.74)*** | 0.46 (0.41, 0.50)*** |  | 0.42 (0.36, 0.47)*** | 0.27 (0.23, 0.30)*** |
| Parent PANAS 4 | 0.30 (0.26, 0.34)*** | 0.14 (0.11, 0.17)*** | 0.20 (0.18, 0.23)*** |  | 0.27 (0.25, 0.29)*** |
| Parent PANAS 5 | 0.55 (0.48, 0.62)*** | 0.23 (0.18, 0.28)*** | 0.38 (0.33, 0.42)*** | 0.74 (0.68, 0.80)*** |  |
| Parent S-DERS 1 | 0.60 (0.57, 0.64)*** | 0.36 (0.34, 0.39)*** | 0.30 (0.27, 0.32)*** | 0.39 (0.36, 0.43)*** | 0.19 (0.17, 0.21)*** |
| Parent S-DERS 2 | 0.67 (0.62, 0.71)*** | 0.52 (0.49, 0.56)*** | 0.36 (0.33, 0.40)*** | 0.30 (0.25, 0.35)*** | 0.19 (0.16, 0.22)*** |
| Parent S-DERS 3 | 0.34 (0.31, 0.37)*** | 0.22 (0.20, 0.24)*** | 0.15 (0.13, 0.17)*** | 0.22 (0.19, 0.25)*** | 0.11 (0.09, 0.12)*** |
| Parent S-DERS 4 | -0.06 (-0.08, -0.03)*** | -0.02 (-0.04, 0.00)* | -0.03 (-0.05, -0.02)*** | -0.09 (-0.11, -0.06)*** | -0.03 (-0.05, -0.02)*** |
| Parent S-DERS 5 | 0.12 (0.08, 0.16)*** | 0.08 (0.05, 0.11)*** | 0.08 (0.06, 0.11)*** | 0.09 (0.05, 0.13)*** | 0.08 (0.06, 0.11)*** |
| Child PANAS 1 | 0.82 (0.66, 0.98)*** | 0.39 (0.28, 0.51)*** | 0.37 (0.26, 0.48)*** | 0.31 (0.16, 0.47)*** | 0.19 (0.10, 0.28)*** |
| Child PANAS 2 | 0.35 (0.30, 0.41)*** | 0.22 (0.17, 0.26)*** | 0.11 (0.07, 0.15)*** | 0.10 (0.05, 0.16)*** | 0.02 (-0.02, 0.05) |
| Child PANAS 3 | 0.40 (0.31, 0.49)*** | 0.23 (0.16, 0.29)*** | 0.18 (0.12, 0.24)*** | 0.14 (0.05, 0.23)** | 0.03 (-0.02, 0.08) |
| Child PANAS 4 | 0.36 (0.26, 0.47)*** | 0.20 (0.13, 0.28)*** | 0.17 (0.10, 0.24)*** | 0.11 (0.01, 0.21)* | 0.02 (-0.04, 0.07) |
| Child PANAS 5 | 0.29 (0.24, 0.34)*** | 0.15 (0.12, 0.19)*** | 0.10 (0.06, 0.13)*** | 0.05 (0.01, 0.10)* | 0.02 (-0.01, 0.05) |
| Child S-DERS 1 | 0.28 (0.24, 0.33)*** | 0.15 (0.11, 0.18)*** | 0.09 (0.06, 0.13)*** | 0.09 (0.05, 0.14)*** | 0.01 (-0.02, 0.03) |
| Child S-DERS 2 | 0.34 (0.29, 0.39)*** | 0.21 (0.17, 0.24)*** | 0.12 (0.08, 0.15)*** | 0.10 (0.05, 0.16)*** | 0.03 (0.00, 0.06) |
| Child S-DERS 3 | 0.30 (0.25, 0.34)*** | 0.17 (0.13, 0.20)*** | 0.09 (0.06, 0.12)*** | 0.12 (0.07, 0.16)*** | 0.01 (-0.01, 0.04) |
| Child S-DERS 4 | 0.26 (0.22, 0.29)*** | 0.17 (0.14, 0.19)*** | 0.09 (0.06, 0.11)*** | 0.11 (0.07, 0.14)*** | 0.02 (-0.01, 0.04) |

* = *p* < 0.05; ** = *p* < 0.01; *** = *p* < 0.001
