# Supplementary material for: Momentary assessment of parent and child emotion regulation to inform the design of a new emotion-focused parenting app
Source: PLoS One. 2025 Jul 3;20(7):e0327179. doi: 10.1371/journal.pone.0327179 (PMC12225822; doi:10.1371/journal.pone.0327179)
Supplement: S12 Table — (DOCX) [file pone.0327179.s012.docx]

**S12 Table. Association of individual child PANAS short survey items with other short survey items.**

| Short survey item | Child PANAS items, *B* (95% CI [*LL, UL*]) | | | | |
| --- | --- | --- | --- | --- | --- |
|  | Item 1 (Depressed) | Item 2 (Angry) | Item 3 (Scared) | Item 4 (Afraid) | Item 5 (Sad) |
| Parent PANAS 1 | 0.08 (0.07, 0.10)*** | 0.29 (0.25, 0.34)*** | 0.13 (0.10, 0.15)*** | 0.09 (0.07, 0.12)*** | 0.31 (0.26, 0.37)*** |
| Parent PANAS 2 | 0.08 (0.06, 0.11)*** | 0.33 (0.27, 0.39)*** | 0.14 (0.10, 0.18)*** | 0.10 (0.06, 0.14)*** | 0.31 (0.24, 0.38)*** |
| Parent PANAS 3 | 0.09 (0.06, 0.12)*** | 0.20 (0.13, 0.28)*** | 0.14 (0.09, 0.18)*** | 0.11 (0.07, 0.15)*** | 0.24 (0.16, 0.32)*** |
| Parent PANAS 4 | 0.04 (0.02, 0.06)*** | 0.10 (0.06, 0.15)*** | 0.06 (0.03, 0.09)*** | 0.05 (0.02, 0.07)*** | 0.10 (0.04, 0.15)*** |
| Parent PANAS 5 | 0.08 (0.05, 0.11)*** | 0.08 (0.00, 0.16) | 0.07 (0.02, 0.12)** | 0.06 (0.01, 0.10)* | 0.10 (0.00, 0.19) |
| Parent S-DERS 1 | 0.08 (0.06, 0.09)*** | 0.26 (0.21, 0.30)*** | 0.12 (0.09, 0.14)*** | 0.08 (0.05, 0.10)*** | 0.31 (0.26, 0.36)*** |
| Parent S-DERS 2 | 0.09 (0.07, 0.11)*** | 0.33 (0.28, 0.39)*** | 0.16 (0.12, 0.19)*** | 0.12 (0.09, 0.15)*** | 0.33 (0.27, 0.39)*** |
| Parent S-DERS 3 | 0.04 (0.03, 0.06)*** | 0.17 (0.13, 0.21)*** | 0.06 (0.04, 0.09)*** | 0.05 (0.03, 0.07)*** | 0.19 (0.15, 0.23)*** |
| Parent S-DERS 4 | -0.01 (-0.02, 0.00) | -0.04 (-0.07, -0.01)** | 0.00 (-0.02, 0.02) | 0.00 (-0.01, 0.02) | -0.07 (-0.11, -0.04)*** |
| Parent S-DERS 5 | 0.03 (0.01, 0.04)*** | 0.06 (0.01, 0.11)** | 0.05 (0.03, 0.08)*** | 0.04 (0.01, 0.06)** | 0.02 (-0.03, 0.07) |
| Child PANAS 1 |  | 0.85 (0.70, 0.99)*** | 0.63 (0.54, 0.72)*** | 0.44 (0.37, 0.52)*** | 0.93 (0.76, 1.10)*** |
| Child PANAS 2 | 0.10 (0.09, 0.12)*** |  | 0.16 (0.13, 0.19)*** | 0.11 (0.08, 0.14)*** | 0.51 (0.46, 0.56)*** |
| Child PANAS 3 | 0.20 (0.17, 0.23)*** | 0.41 (0.33, 0.49)*** |  | 0.71 (0.68, 0.74)*** | 0.64 (0.55, 0.74)*** |
| Child PANAS 4 | 0.17 (0.14, 0.21)*** | 0.35 (0.26, 0.45)*** | 0.88 (0.85, 0.92)*** |  | 0.71 (0.60, 0.81)*** |
| Child PANAS 5 | 0.09 (0.07, 0.10)*** | 0.39 (0.35, 0.43)*** | 0.18 (0.16, 0.21)*** | 0.16 (0.14, 0.19)*** |  |
| Child S-DERS 1 | 0.09 (0.07, 0.10)*** | 0.46 (0.43, 0.50)*** | 0.18 (0.16, 0.21)*** | 0.16 (0.14, 0.18)*** | 0.60 (0.56, 0.64)*** |
| Child S-DERS 2 | 0.10 (0.08, 0.11)*** | 0.47 (0.43, 0.51)*** | 0.22 (0.19, 0.25)*** | 0.19 (0.16, 0.21)*** | 0.63 (0.59, 0.68)*** |
| Child S-DERS 3 | 0.07 (0.06, 0.09)*** | 0.44 (0.40, 0.47)*** | 0.17 (0.14, 0.19)*** | 0.14 (0.12, 0.16)*** | 0.50 (0.46, 0.54)*** |
| Child S-DERS 4 | 0.06 (0.05, 0.07)*** | 0.30 (0.27, 0.34)*** | 0.13 (0.11, 0.15)*** | 0.11 (0.09, 0.13)*** | 0.39 (0.35, 0.42)*** |

* = *p* < 0.05; ** = *p* < 0.01; *** = *p* < 0.001
