# Supplementary material for: Momentary assessment of parent and child emotion regulation to inform the design of a new emotion-focused parenting app
Source: PLoS One. 2025 Jul 3;20(7):e0327179. doi: 10.1371/journal.pone.0327179 (PMC12225822; doi:10.1371/journal.pone.0327179)
Supplement: S13 Table — (DOCX) [file pone.0327179.s013.docx]

**S13 Table. Association of individual parent S-DERS short survey items with other short survey items.**

| Short survey item | Parent S-DERS items, *B* (95% CI [*LL, UL*]) | | | | |
| --- | --- | --- | --- | --- | --- |
|  | Item 1 | Item 2 | Item 3 | Item 4 | Item 5 |
| Parent PANAS 1 | 0.63 (0.60, 0.67)*** | 0.39 (0.36, 0.42)*** | 0.55 (0.50, 0.59)*** | -0.14 (-0.20, -0.07)*** | 0.12 (0.08, 0.16)*** |
| Parent PANAS 2 | 0.69 (0.64, 0.74)*** | 0.57 (0.53, 0.61)*** | 0.65 (0.58, 0.72)*** | -0.11 (-0.19, -0.02)* | 0.16 (0.10, 0.22)*** |
| Parent PANAS 3 | 0.71 (0.65, 0.76)*** | 0.48 (0.44, 0.52)*** | 0.53 (0.46, 0.61)*** | -0.18 (-0.28, -0.08)*** | 0.18 (0.12, 0.25)*** |
| Parent PANAS 4 | 0.46 (0.42, 0.50)*** | 0.20 (0.17, 0.23)*** | 0.42 (0.37, 0.47)*** | -0.22 (-0.29, -0.16)*** | 0.11 (0.06, 0.15)*** |
| Parent PANAS 5 | 0.62 (0.55, 0.69)*** | 0.35 (0.30, 0.40)*** | 0.52 (0.43, 0.61)*** | -0.24 (-0.35, -0.13)*** | 0.26 (0.19, 0.33)*** |
| Parent S-DERS 1 |  | 0.47 (0.44, 0.49)*** | 0.68 (0.63, 0.72)*** | -0.22 (-0.28, -0.16)*** | 0.20 (0.16, 0.25)*** |
| Parent S-DERS 2 | 0.82 (0.78, 0.87)*** |  | 0.81 (0.75, 0.87)*** | -0.17 (-0.25, -0.08)*** | 0.23 (0.18, 0.29)*** |
| Parent S-DERS 3 | 0.44 (0.41, 0.46)*** | 0.30 (0.27, 0.32)*** |  | -0.10 (-0.15, -0.05)*** | 0.10 (0.07, 0.14)*** |
| Parent S-DERS 4 | -0.10 (-0.12, -0.07)*** | -0.04 (-0.06, -0.02)*** | -0.07 (-0.10, -0.03)*** |  | 0.06 (0.03, 0.09)*** |
| Parent S-DERS 5 | 0.20 (0.16, 0.24)*** | 0.13 (0.10, 0.16)*** | 0.16 (0.11, 0.21)*** | 0.14 (0.07, 0.20)*** |  |
| Child PANAS 1 | 0.78 (0.62, 0.95)*** | 0.54 (0.40, 0.67)*** | 0.67 (0.46, 0.88)*** | -0.23 (-0.48, 0.02) | 0.25 (0.07, 0.42)** |
| Child PANAS 2 | 0.32 (0.26, 0.38)*** | 0.26 (0.22, 0.31)*** | 0.32 (0.25, 0.40)*** | -0.12 (-0.21, -0.03)** | 0.08 (0.01, 0.14)* |
| Child PANAS 3 | 0.37 (0.27, 0.46)*** | 0.31 (0.23, 0.39)*** | 0.31 (0.19, 0.43)*** | 0.01 (-0.13, 0.16) | 0.15 (0.05, 0.25)** |
| Child PANAS 4 | 0.28 (0.17, 0.39)*** | 0.29 (0.20, 0.38)*** | 0.29 (0.15, 0.42)*** | -0.01 (-0.17, 0.15) | 0.11 (0.00, 0.22) |
| Child PANAS 5 | 0.31 (0.26, 0.36)*** | 0.21 (0.17, 0.25)*** | 0.29 (0.22, 0.35)*** | -0.18 (-0.26, -0.11)*** | 0.01 (-0.04, 0.06) |
| Child S-DERS 1 | 0.30 (0.26, 0.35)*** | 0.22 (0.18, 0.26)*** | 0.29 (0.23, 0.34)*** | -0.17 (-0.24, -0.10)*** | 0.06 (0.01, 0.11)* |
| Child S-DERS 2 | 0.33 (0.27, 0.38)*** | 0.29 (0.25, 0.33)*** | 0.32 (0.25, 0.39)*** | -0.14 (-0.22, -0.06)*** | 0.09 (0.03, 0.15)** |
| Child S-DERS 3 | 0.30 (0.25, 0.34)*** | 0.23 (0.19, 0.27)*** | 0.30 (0.25, 0.36)*** | -0.11 (-0.18, -0.05)** | 0.08 (0.03, 0.13)** |
| Child S-DERS 4 | 0.27 (0.23, 0.31)*** | 0.21 (0.18, 0.24)*** | 0.27 (0.22, 0.31)*** | -0.13 (-0.19, -0.07)*** | 0.05 (0.01, 0.09)* |

* = *p* < 0.05; ** = *p* < 0.01; *** = *p* < 0.001
