# Supplementary material for: Momentary assessment of parent and child emotion regulation to inform the design of a new emotion-focused parenting app
Source: PLoS One. 2025 Jul 3;20(7):e0327179. doi: 10.1371/journal.pone.0327179 (PMC12225822; doi:10.1371/journal.pone.0327179)
Supplement: S14 Table — (DOCX) [file pone.0327179.s014.docx]

**S14 Table. Association of individual child S-DERS short survey items with other short survey items.**

| Short survey item | Child S-DERS items, *B* (95% CI [*LL, UL*]) | | | |
| --- | --- | --- | --- | --- |
|  | Item 1 | Item 2 | Item 3 | Item 4 |
| Parent PANAS 1 | 0.37 (0.32, 0.43)*** | 0.34 (0.28, 0.39)*** | 0.41 (0.35, 0.47)*** | 0.46 (0.39, 0.53)*** |
| Parent PANAS 2 | 0.36 (0.28, 0.44)*** | 0.39 (0.32, 0.46)*** | 0.43 (0.34, 0.51)*** | 0.57 (0.48, 0.67)*** |
| Parent PANAS 3 | 0.28 (0.19, 0.38)*** | 0.26 (0.18, 0.34)*** | 0.26 (0.17, 0.35)*** | 0.36 (0.25, 0.46)*** |
| Parent PANAS 4 | 0.16 (0.10, 0.22)*** | 0.14 (0.09, 0.19)*** | 0.20 (0.13, 0.26)*** | 0.22 (0.15, 0.29)*** |
| Parent PANAS 5 | 0.06 (-0.05, 0.17) | 0.14 (0.04, 0.23)** | 0.10 (-0.01, 0.21) | 0.12 (-0.01, 0.25) |
| Parent S-DERS 1 | 0.38 (0.33, 0.43)*** | 0.32 (0.27, 0.37)*** | 0.40 (0.35, 0.46)*** | 0.48 (0.42, 0.55)*** |
| Parent S-DERS 2 | 0.44 (0.37, 0.51)*** | 0.43 (0.37, 0.49)*** | 0.49 (0.42, 0.56)*** | 0.61 (0.53, 0.70)*** |
| Parent S-DERS 3 | 0.24 (0.20, 0.29)*** | 0.21 (0.17, 0.25)*** | 0.27 (0.22, 0.31)*** | 0.32 (0.26, 0.37)*** |
| Parent S-DERS 4 | -0.08 (-0.12, -0.04)*** | -0.05 (-0.08, -0.01)** | -0.06 (-0.10, -0.02)** | -0.09 (-0.14, -0.04)*** |
| Parent S-DERS 5 | 0.08 (0.03, 0.14)** | 0.09 (0.04, 0.14)*** | 0.10 (0.05, 0.16)*** | 0.10 (0.03, 0.17)** |
| Child PANAS 1 | 1.13 (0.94, 1.31)*** | 0.94 (0.79, 1.10)*** | 0.97 (0.78, 1.16)*** | 1.04 (0.82, 1.26)*** |
| Child PANAS 2 | 0.72 (0.66, 0.77)*** | 0.55 (0.50, 0.60)*** | 0.71 (0.66, 0.77)*** | 0.67 (0.60, 0.74)*** |
| Child PANAS 3 | 0.78 (0.68, 0.88)*** | 0.66 (0.57, 0.75)*** | 0.71 (0.60, 0.82)*** | 0.77 (0.65, 0.89)*** |
| Child PANAS 4 | 0.84 (0.72, 0.95)*** | 0.71 (0.61, 0.81)*** | 0.75 (0.63, 0.87)*** | 0.79 (0.65, 0.93)*** |
| Child PANAS 5 | 0.74 (0.69, 0.78)*** | 0.57 (0.53, 0.61)*** | 0.64 (0.59, 0.69)*** | 0.66 (0.60, 0.72)*** |
| Child S-DERS 1 |  | 0.69 (0.67, 0.72)*** | 0.75 (0.71, 0.79)*** | 0.70 (0.65, 0.75)*** |
| Child S-DERS 2 | 0.93 (0.89, 0.97)*** |  | 0.90 (0.86, 0.94)*** | 0.87 (0.81, 0.92)*** |
| Child S-DERS 3 | 0.71 (0.67, 0.74)*** | 0.64 (0.61, 0.67)*** |  | 0.82 (0.77, 0.86)*** |
| Child S-DERS 4 | 0.50 (0.46, 0.54)*** | 0.46 (0.43, 0.49)*** | 0.61 (0.58, 0.65)*** |  |

* = *p* < 0.05; ** = *p* < 0.01; *** = *p* < 0.001
